# Probing the Biosafety of Implantable Artificial Secretory Granules for the Sustained Release of Bioactive Proteins

**DOI:** 10.1021/acsami.3c08643

**Published:** 2023-08-10

**Authors:** Patricia Álamo, Eloi Parladé, Marianna T. P. Favaro, Alberto Gallardo, Rosa Mendoza, Luís C. S. Ferreira, Nerea Roher, Ramón Mangues, Antonio Villaverde, Esther Vázquez

**Affiliations:** † Institut d’Investigació Biomèdica Sant Pau (IIB SANT PAU), 08041 Barcelona, Spain; ‡ Josep Carreras Leukaemia Research Institute (IJC), 08916 Badalona, Spain; § CIBER de Bioingeniería, Biomateriales y Nanomedicina (CIBER-BBN, ISCIII), Universitat Autònoma de Barcelona, 08193 Bellaterra, Spain; ∥ Institut de Biotecnologia i de Biomedicina, Universitat Autònoma de Barcelona, 08193 Bellaterra, Spain; ⊥ Instituto de Ciências Biomédicas, Universidade de São Paulo, São Paulo 05508-000, Brazil; # Department of Pathology, Hospital de la Santa Creu i Sant Pau, 08025 Barcelona, Spain; ∇ Department of Cell Biology, Animal Physiology and Immunology, Universitat Autònoma de Barcelona, 08193 Bellaterra, Spain; ○ Departament de Genètica i de Microbiologia, Universitat Autònoma de Barcelona, 08193 Bellaterra, Spain

**Keywords:** recombinant protein, protein engineering, self-assembling, protein materials, drug delivery

## Abstract

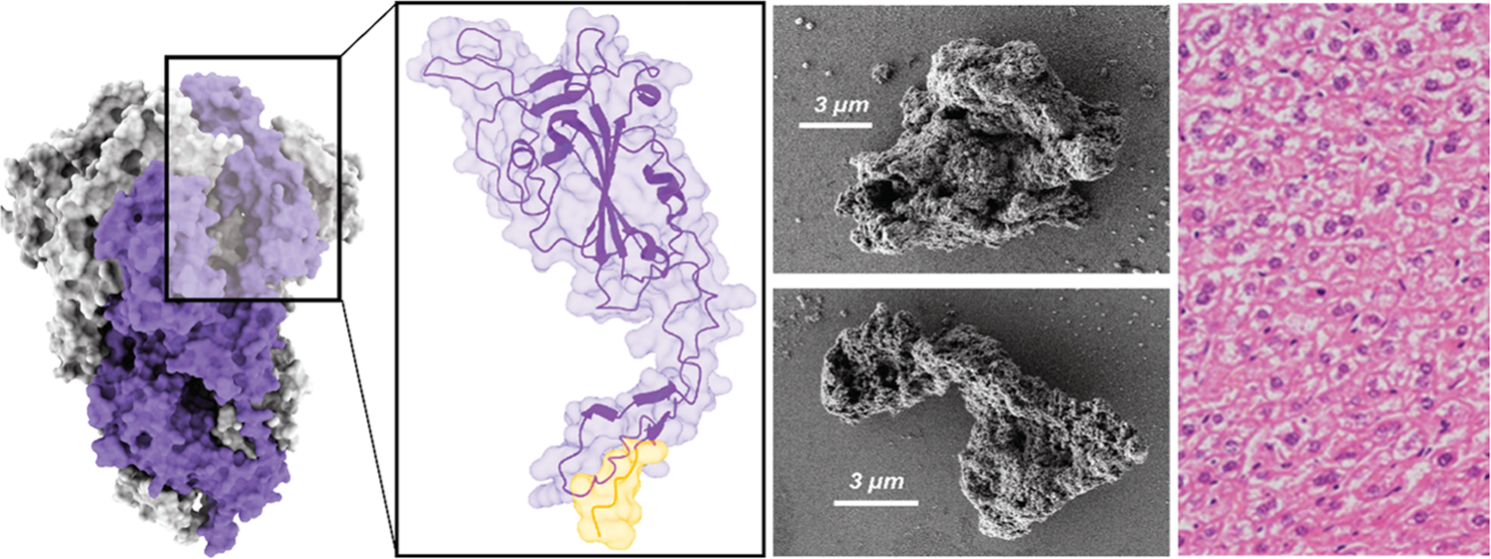

Among bio-inspired protein materials, secretory protein microparticles are of clinical interest as self-contained, slow protein delivery platforms that mimic secretory granules of the human endocrine system, in which the protein is both the drug and the scaffold. Upon subcutaneous injection, their progressive disintegration results in the sustained release of the building block polypeptides, which reach the bloodstream for systemic distribution and subsequent biological effects. Such entities are easily fabricated *in vitro* by Zn-assisted cross-molecular coordination of histidine residues. Using cationic Zn for the assembly of selected pure protein species and in the absence of any heterologous holding material, these granules are expected to be nontoxic and therefore adequate for different clinical uses. However, such presumed biosafety has not been so far confirmed and the potential protein dosage threshold not probed yet. By selecting the receptor binding domain (RBD) from the severe acute respiratory syndrome coronavirus 2 (SARS-CoV-2) spike protein as a model protein and using a mouse lab model, we have explored the toxicity of RBD-made secretory granules at increasing doses up to ∼100 mg/kg of animal weight. By monitoring body weight and biochemical blood markers and through the histological scrutiny of main tissues and organs, we have not observed systemic toxicity. Otherwise, the bioavailability of the material was demonstrated by the induction of specific antibody responses. The presented data confirm the intrinsic biosafety of artificial secretory granules made by recombinant proteins and prompt their further clinical development as self-contained and dynamic protein reservoirs.

## Introduction

The potential toxicity of materials to be in contact with biological interfaces is a great matter of concern in diverse clinical fields.^1−8^ In this context, novel biodegradable and biomimetic materials are under continuous exploration, tailoring, and development to avoid undesired side damage in the patient.^9−13^ Aiming at the delivery of bioactive proteins, including drugs and antigens, protein-based constructs benefit from the intrinsic lack of toxicity of polypeptides as universal biomacromolecules. This is in addition to their functional and structural versatility that can be adjusted by rather simple genetic engineering.^14−17^ Among emerging protein materials, those based on self-assembling architectonic principles allow the fabrication of supramolecular entities with increasing levels of complexity from nano to microscales. The coordination of cationic Zn with histidine (his)-rich peptides displayed on selected proteins allows their controlled cross-molecular interactions,^18−20^ resulting in nanoparticles or microparticles depending on the metal/his ratio used for coordination.^21^ While nanoparticles are useful as nanostructured drugs or drug vehicles,^22,23^ microparticles show an intriguing amyloidal architecture and slow protein-leaking properties.^24−26^ This type of protein depot resembles, in both structure and function, secretory granules of peptide hormones occurring in the mammalian endocrine system^27−30^ in which the proteins are self-contained as mechanically stable, amyloid-like protein-only structures.^28,31^ Regarding both their rough and irregular surfaces and their intrinsic molecular organization, they also resemble bacterial inclusion bodies^32^ that also show intriguing protein-releasing properties.^33−35^ Secretory granules and bacterial inclusion bodies are considered examples of functional amyloids in nature.^27,30,36−39^

Such synthetic microscale secretory granules have been observed as long-term dynamic reservoirs of functional proteins^24,40^ that are made available to the bloodstream during the progressive disintegration of the granules after subcutaneous administration.^26^ The disintegration process is progressive, and it results in the sustained leakage of the forming protein.^26^ This event is probably mediated by a physiological dilution of Zn cations from the complexes that result in the separation of monomeric or oligomeric polypeptides from the bulk material. Such artificial granules, fabricated in the laboratory by exploiting the interactivity between cationic metals and histidine residues, differ from other slow-release platforms in that the protein is self-contained in the particle, in the absence of any scaffolding, porous, or matrix-like agents to hold it.^41^ Using several *in vitro* and *in vivo* models, these emerging materials have been proved useful and promising in the sustained delivery of bioactive proteins in different fields, including regenerative medicine,^42^ cancer treatment,^24^ or as drugs for neurodegenerative disorders.^43^ Despite their potential applicability as a generic platform with a wide spectrum of applications in clinics, the potential toxicity of the system, when using elevated protein amounts, remains unexplored, and the upper threshold dosage has not been established yet. The usual working doses of such protein-releasing materials, proven useful for many applications, are expected to contain Zn amounts below the daily recommended intake,^21^ but specific conditions and applications involving elevated local amounts of a given polypeptide drug might require high dosage. In the present study, we have subcutaneously administered increasing amounts of microscale secretory granules in a mouse model to challenge the biological limits of the system and to explore potential adverse effects. The obtained results reveal the absence of systemic toxicity when up to ∼100 mg/kg of animal weight has been administered, while the results confirm the bioavailability of the protein after administration. The whole set of data endorses artificial secretory granules as promising biosafe drug delivery systems that in their development toward clinics should not pose regulatory issues linked to toxicity.

## Materials and Methods

### Protein Synthesis and Preparation of Granules

The gene encoding eRBD-H6, an extended version of the receptor binding domain (RBD) from the severe acute respiratory syndrome coronavirus 2 (SARS-CoV-2) spike protein,^44^ was inserted into pET22b and expressed in *Escherichia coli* BL21 DE3 (Novagen-Merck, Darmstadt, Germany). Culture samples were routinely analyzed using sodium dodecyl sulfate–polyacrylamide gel electrophoresis (SDS-PAGE) and Western blot with anti-His mouse monoclonal antibodies to confirm protein production. Cell pellets were harvested and resuspended in 50 mM Tris, pH 8.0, in the presence of protease inhibitors (cOmplete EDTA-Free, Roche, Basel, Switzerland). eRBD-H6 was solubilized from inclusion bodies by a previously described purification protocol.^44^ In summary, cell pellets were sonicated (8 min at 40% amplitude, 1 s ON, 4 s OFF, Branson digital sonifier, MO) and centrifuged (8228*g*, 30 min). The sediment, including inclusion bodies, was washed and resuspended in a solubilization buffer (1 mM EDTA, 15 mM DTT, 6 M guanidine hydrochloride) for protein refolding (0.18 mM EDTA, 0.5 M l-arginine, 1.9 mM reduced glutathione, 0.9 mM oxidized glutathione, 2 M urea in 20 mM phosphate buffer, pH 8.0). Finally, the protein was dialyzed against a battery of intermediate buffers for a soft transition to the storage buffer (75 mM l-arginine, 233 mM sucrose, 20 mM phosphate buffer, pH 8.0). The integrity of the refolded protein was assessed via SDS-PAGE and matrix-assisted laser desorption/ionization–time of flight (MALDI-TOF).

The *in vitro* fabrication of microparticles out of refolded protein was based on a modified version of a previously described method.^22^ Briefly, the microscale granules were formed by adding a molar excess of ionic Zn to protein samples, relative to the number of histidine residues in the H6 tag. Such a tag, placed at the carboxy terminus, was then used for a cross-molecular Zn-mediated anchorage between homologous polypeptides.^18^ Specifically, 30, 100, 300, or 1000 μg of soluble protein was mixed with ZnCl_2_ in a molar excess ratio of 1:300 (protein/cation) in potassium–sodium phosphate-buffered saline with a pH value of 7.4, within a physiological range and optimal for *in vivo* studies, with a final protein concentration of 1 mg/mL in a final volume of 100 μL. After mixing the components and allowing them to react for 10 min, the samples were centrifuged at 15,000*g* for 15 min to separate the microgranules, which remain insoluble and tend to sediment from the unreacted soluble protein in the supernatant. High-resolution electron microscopy images of the microparticles were obtained using field emission scanning electron microscopy (FESEM Zeiss Merlin, Oberkochen, Germany) operating at 1 kV and equipped with a high-resolution secondary electron detector.

### *In Vivo* Experiments

Experiments involving animals were conducted through procedures according to the European Council directives and approved by the Animal Ethics Committee at the Hospital de la Santa Creu i Sant Pau (procedure 9721). Male and female BALB/c mice, aged 6–8 weeks and weighing between 16–27 g, were obtained from Charles River (L’Arbresle, France) and housed in pathogen-free conditions. The administration regimen included 1 or 2 repeated doses of different amounts of microgranules (1000, 300, 100, or 30 μg) or phosphate-buffered saline (PBS) in the control groups. The mice were randomly allocated in each group, with doses administered on days 0 and 21. The administration was done subcutaneously in the lumbar region of the mice using a pellet of protein antigen granules suspended in 150 μL of PBS buffer. Mice were weighed 3 times a week to monitor for any weight loss.

### Humoral Response and Histology

On day 35 after the initial injection, blood samples were collected from the mice to measure the humoral response to the protein antigen. The collected sera were analyzed for antibody levels using the Recombivirus Mouse Anti SARS-CoV-2 (COVID) Spike 1 RBD IgG ELISA Kit (RV-405420 α Diagnostics, San Antonio, Texas) following the manufacturer′s instructions. Mice were euthanized after day 35, and their liver, kidney, and spleen were resected. The tissues were fixed in 4% paraformaldehyde, embedded in paraffin, sectioned, and stained with hematoxylin and eosin (H&E) for histological analysis, which was performed by two independent observers using an Olympus BX53 microscope.

### Biochemistry

The liver function and renal function were evaluated by measuring the serum levels of aspartate aminotransferase (AST), alanine aminotransferase (ALT), creatinine (CRE), and uric acid (UA) using commercial kits (20764949322, 20764957322, 03183807190, and 04810716190, respectively, Roche, Basel, Switzerland) adapted for a COBAS 6000 autoanalyzer (Roche, Basel, Switzerland).

### Statistical Analysis

Data are presented as mean ± standard deviation. Statistical analyses were performed using GraphPad Prism version 8.0.2 (GraphPad Software, San Diego, California). Differences between groups were analyzed using a one-way ANOVA or Kruskal–Wallis test, followed by Dunnett’s or Dunn’s multiple comparisons test, respectively. Statistical significance levels were set at *p* < 0.05 (*) and *p* < 0.001(***).

## Results

**Figure 1:**
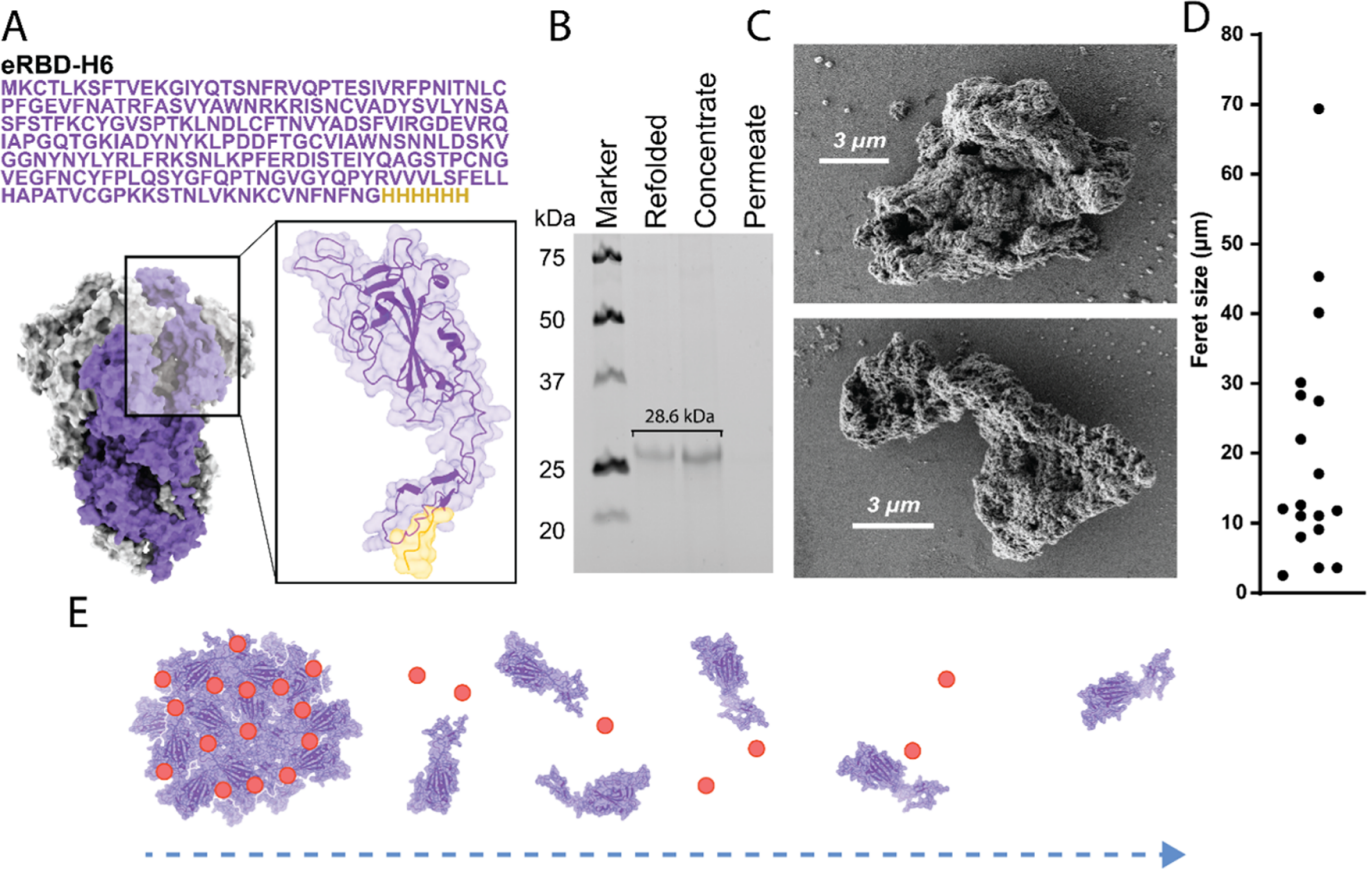
Composition of protein secretory granules. (A) eRBD-H6 amino acid sequence including the H6 tag used for both purification and metal-based clustering. At the bottom, the molecular model of the trimeric spike protein (PDB: 6VXX) is shown. The inset shows the tridimensional structure of the eRBD-H6 construct as predicted by AlphaFold2 with the H6 tag solvent-exposed region highlighted in yellow. Additional structural details can be found elsewhere.^44^ (B) SDS-PAGE showing eRBD-H6 resulting from IB protein refolding and from the concentrate version ready for clustering. The apparent mobility was consistent with the expected molecular mass of the protein, also confirmed by mass spectrometry (28.6 kDa, not shown). (C) A FESEM micrograph shows the detail of two microgranules randomly picked among those occurring in the main size range (around 10 μm, see panel D). (D) Size distribution of the material population upon clustering determined by FESEM image analysis. (E) General functioning of the self-contained, self-disintegrating protein depots. Polypeptides (purple) are clustered together by the coordination of divalent Zn cations (red balls) with histidine-rich end terminal tags into mechanically stable microparticles (left). Upon *in vivo* administration, these materials slowly disintegrate along time (dashed blue line), releasing monomeric or oligomeric forms of the protein (right, linked to the loss of ionic Zn from the material), which are then available for desired functionalities.

Protein microgranules made of the RBD domain from the SARS-CoV-2 spike protein (extended version, eRBD, Figure 1A^44^) were fabricated with pure recombinant proteins produced in *E. coli* (Figure 1B). The formation of protein clusters was triggered by the addition of ZnCl_2_ to protein solutions that resulted in discrete microparticles (Figure 1C) with an average size of 20.3 ± 17.0 μm (*n* = 18, Figure 1D) and a median of 12.4 μm. As determined in several model proteins of different functional and structural complexities, including eRBD,^24,26,40,42−45^ such microparticles are self-disintegrating under physiological conditions, probably by a progressive loss of Zn under dilution. This fact results in a consequent time-prolonged leakage of monomeric or oligomeric forms of the protein (Figure 1E) that are properly folded and functional.^24^ Then, the material, once administered *in vivo*, acts as an intriguing protein delivery platform in which the building block polypeptides are self-contained and finally self-delivered from those dynamic, protein-only depots.

**Figure 2:**
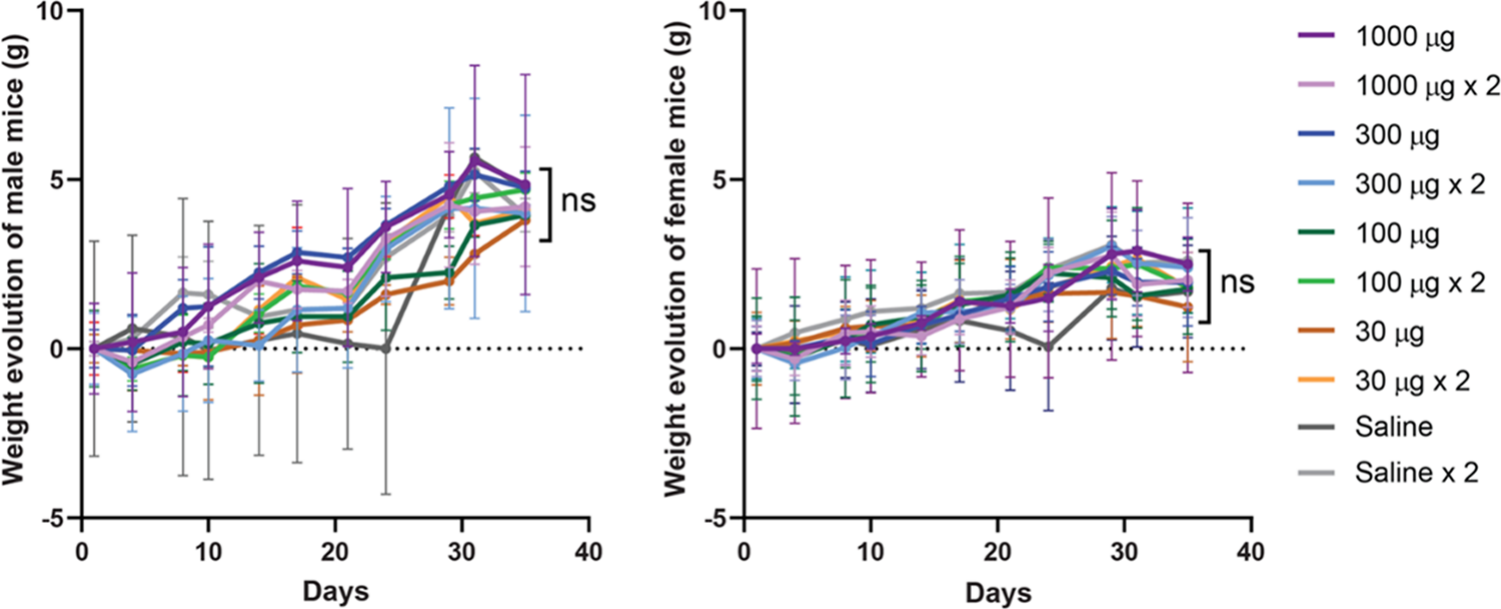
Body weight evolution of experimental animal groups administered with secretory granules. Body weight tracking of male (A) and female (B) mice groups upon administration of different amounts of protein microgranules in either a single or a double (×2) dose. All weight measurements were individually normalized by the initial weight on day 0, with values representing the increase in body weight along time. Days 0 and 21 are the administration times. Differences between group pairs were seen as not significant (ns) in any case.

Different amounts of the granules were subcutaneously administered in a mouse model, in both female and male groups. In one set of groups, one single protein bolus was administered at day 0, and in another, a second bolus was administered three weeks later. In that case, not only were the doses doubled but we also reproduced the 1–3 weeks of administration regimes for enzyme replacement therapies^46,47^ (as a model of conditions treatable by protein administration) or conventional vaccination to amplify any potential inflammatory or immunotoxic response. Any external sign of inflammation or irritation at the administration site was observed upon injection. Also, the whole-body weight of the animals was monitored for 35 days. As observed, the administration of protein did not significantly affect the gain of body weight that was progressive in all of the groups and during the whole experimental time (Figure 2). The weight gain of the groups receiving two doses was comparable with that shown by saline-injected animal groups. This first set of data suggested the absence of toxicity linked to the administered protein as formulated in the form of microgranules even at high doses (2000 μg). For 20 g-weight animals, such an upper dose represents approximately 100 mg of protein/kg of body weight, far over any envisaged clinical use in humans.

**Figure 3:**
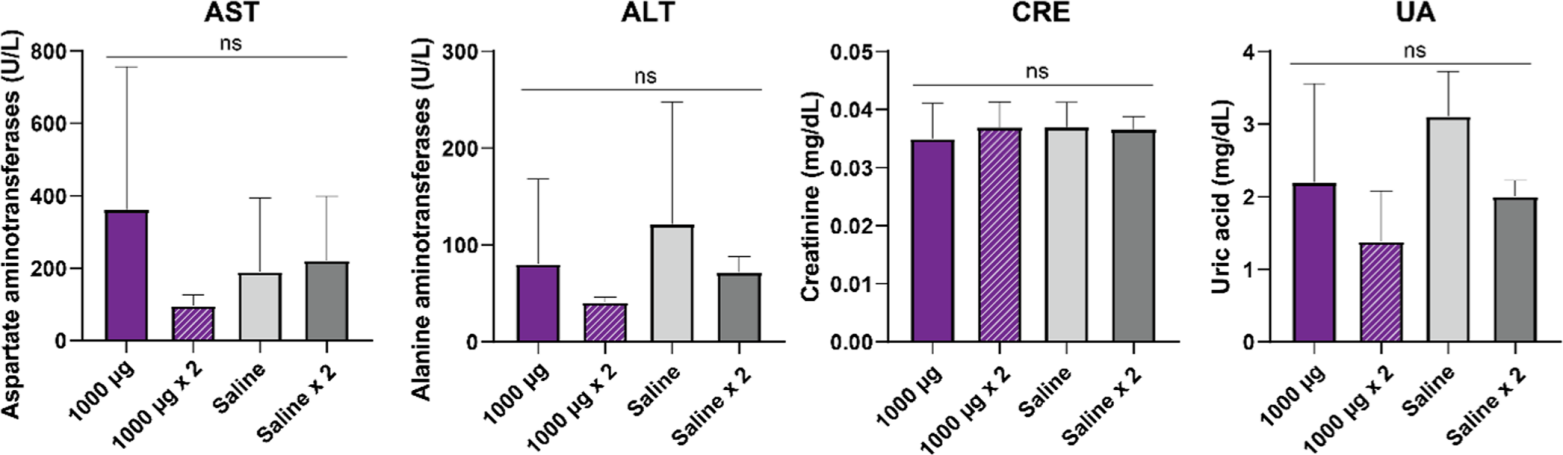
Analysis of blood biochemical markers. Determination of AST, ALT, CRE, and UA in animals administered with microscale secretory granules. Blood samples were collected at day 35. Differences between equivalent groups (treated and nontreated) were seen as not significant (ns) in all cases.

**Figure 4:**
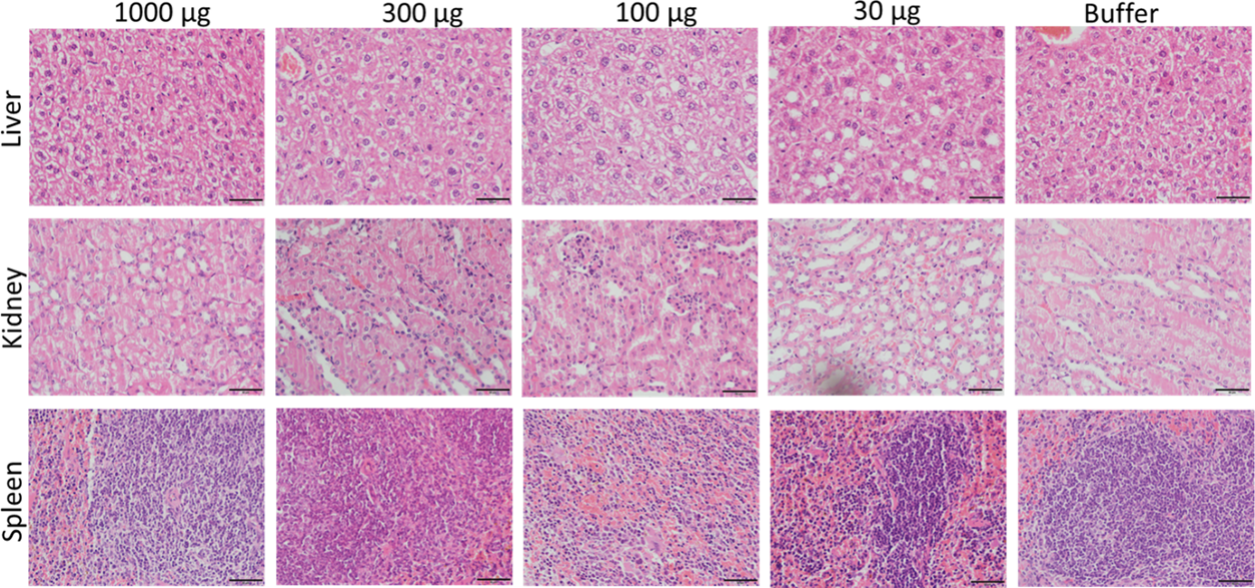
Histology of main organs in treated animals. Representative hematoxylin and eosin stain of the liver, kidney, and spleen, collected 35 days after the administration of different doses of secreting granules. Bar: 50 μm.

Aiming to assess the impact of secretory granules on liver and kidney function, we measured biochemical markers in blood (AST, ALT, CRE, and UA) at the end of the experimental time in those mice groups administered with the highest protein amounts (*i.e.*, one or two doses of 1000 μg) to evaluate damage in main organs. Data presented in Figure 3 showed no statistically significant alterations in those markers compared to the saline-administered control. In any case, a second dose enhanced the level of those markers that in general were slightly lower (although at a nonsignificant level) upon such second administration. While a significant increase in these markers would be associated with hepatic or renal damage, the slight tendency toward a reduction of AST and ALT in treated animals cannot be linked to any condition, as low levels of these markers are in general considered protective.^48^ In any case, all of the obtained values are within those considered normal for healthy BALB/c mice, namely, between 55–352 U/L for AST and between 41–131 U/L for ALT (www.criver.com; retrieved on July 10, 2023). Furthermore, the absence of organ toxicity was finally confirmed through the histopathological analysis of the spleen, kidney, and liver (Figure 4).

**Figure 5:**
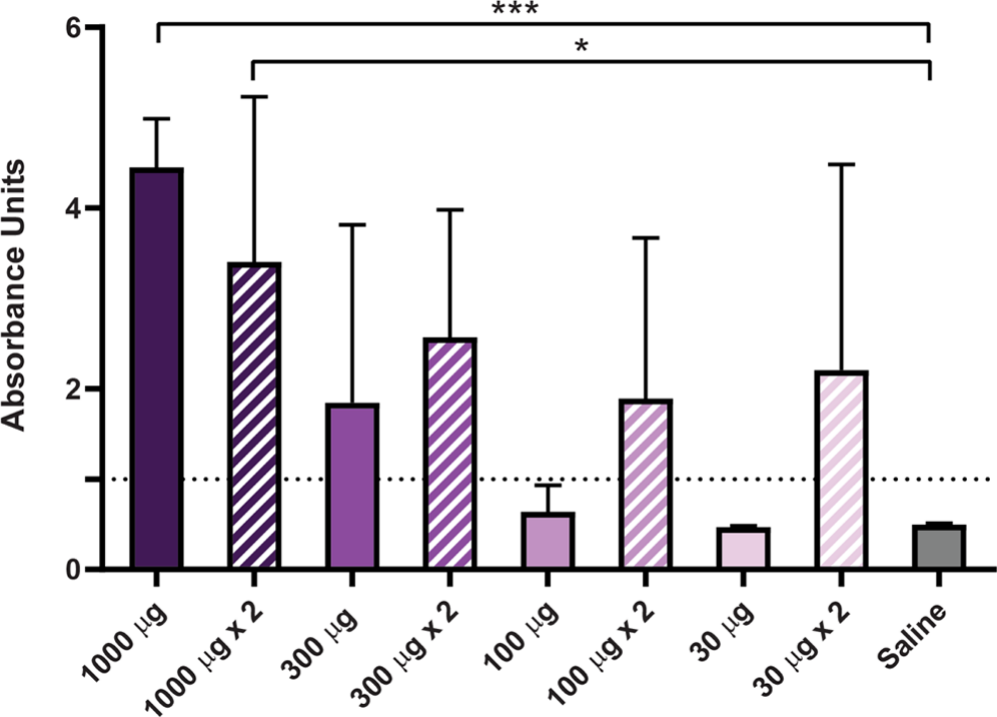
Humoral immune response triggered against subcutaneously administered protein granules. Blood samples were collected 35 days after administration and tested in an anti SARS-CoV-2 ELISA IgG kit.

In previous studies, the protein leakage from artificial secretory granules obtained by Zn-his coordination^24,26^ and in particular from eRDB-H6 microgranules^44^ had been robustly described. To fully confirm that the absence of toxicity described above occurred in a truly secretory system, the bioavailability of the protein was assessed through the triggered immune response. Indeed, anti-protein antibodies were detected in administered animals, with a response generically tending to be higher at higher doses (Figure 5). It must be noted that the second administration of the protein material only enhanced the antibody titer moderately. Altogether, these data confirmed the effective protein release from granules in all tested doses while also suggesting that immunotoxicities or exacerbated immune responses did not occur.

## Discussion

In conventional nanoparticle-based drug delivery, the need for a drug vehicle is a matter of concern as it contributes to the system with a bulk of nontherapeutic material that usually represents the majority fraction of the whole drug complex mass.^49^ Such a carrier system might display individual and environmental toxicities. It represents additional production costs, and it also adds important complexity to the formulation process and regulatory challenges to the approval for clinical uses.^49^ In this regard, the concept of self-assembling, self-targeted drugs lacking heterologous vehicles has gained interest in nanobiotechnology.^50^ Using bioactive proteins organized as tumor-targeted nanoparticles, this vehicle-free principle has been fully demonstrated in several models of human cancer,^51−53^ and such a concept can be extended to any bioactive protein usable in clinics, irrespective of the function to be performed in the body. This includes antimicrobial peptides, cytotoxic proteins, targeting agents, imaging agents, immunomodulators, antigens, enzymes, hormones, and growth factors, among others.

On the other hand, the development of slow-release systems, for both chemicals and proteins, faces a similar bottleneck. Novel delivery systems based on the sustained release of the payload agent are highly desired to reduce its level oscillations and to keep constant concentrations at the site of action.^54,55^ Porous and hollowed materials, hydrogels, and different polymeric matrices are among the most explored holding systems for time-prolonged release.^56−61^ In them, the active molecule is stored for further release under physiological conditions. Upon administration, such materials are expected to be mechanically and chemically stable and biologically functional in contact with organic tissues during prolonged periods. Toxicity, then, is again a matter of concern.^62^

In contrast to such holder–drug dual systems, functional amyloids in nature are efficient protein storage systems in which the functional polypeptides are self-clustered together as microgranules with the assistance of cationic metals.^27−29,36,37,63^ The disassembling of these materials is progressive, and it involves the leakage of individual polypeptides by metal chelation or dilution (Figure 1E). Therefore, the protein is the functional agent and also the building block of a mechanically stable structure that hosts itself. This mechanism is the basis of the human endocrine system that stores and releases a diversity of peptide hormones.^28−30^ Many secretory hormones from the mammalian endocrine system form functional amyloids in secretory granules.^27−31^ Importantly, irrespective of their sequence and structure, they aggregate as amyloid fibrils.^28,30^ Thus, protein aggregation and amyloid formation follows a polymerization mechanism where protein or peptide species slowly associate to form aggregation competent nuclei. These cores further recruit the monomeric counterpart to grow into mature amyloid fibrils, eventually reaching a steady-state equilibrium between the fibrils and monomeric proteins.^64^ Based on this equilibrium, the protein storage as an amyloid depot is also able to release the functional monomeric proteins upon dilution.^64^

Mimetic versions of these natural systems have been recently developed, which has resulted in promising delivery systems, fully functional and operational in different experimental settings *in vitro* and *in vivo.*(24,26,42,65) The protein is then self-contained *in vitro* when exposed to cationic Zn and self-delivered *in vivo*, upon administration, when the metal is diluted in the physiological medium. Consequently, these granules follow a similar mechanism as described above for the secretory hormones; thus, sustained release of the building block bioactive proteins may happen because of the progressive disintegration of the amyloids to reach a systemic distribution and achieve the subsequent biological effects. The aggregating polypeptides are then, at the same time, the bioactive agents and the mechanically stable scaffolds that act as a dynamic protein depot. Although the platform itself is progressively understood, becoming suited to specific tailoring, toxicity remains an unexplored issue. Even if unexpected, the possibility of undesired side effects linked to high local concentrations of foreign protein needs to be evaluated before further development. In this regard, the evolution of the whole-body weight (Figure 2) and the histological analysis of main organs (Figure 4) revealed the absence of toxicity, even when the dose of the administered protein (in a granular format) is up to ∼2 mg. This amount, for a reference 20 g animal, represents 100 mg/kg of body weight, which is much beyond the employed doses of therapeutic proteins. The dosage of protein drugs is usually akin to body weight.^66,67^ As a paradigmatic example, in enzyme replacement therapies, protein doses usually range between 0.2 and 20 mg/kg, given systemically every 1–2 weeks.^47^

The moderate antibody response triggered against the granulated model protein (Figure 5) indicates bioavailability of the building block, but it also suggests the absence of a potent immune reaction that could derive into immunopathogenic damage. Importantly, the potential immunotoxicity of therapeutic proteins, especially in aggregated forms, is a generic matter of worry that relies on mechanisms not completely understood.^68,69^ Such adverse events are mostly expected during systemic administration in blood,^70,71^ a fact that can dramatically reduce the therapeutic or in general the biological impact of the protein by triggering protein-binding antibodies.^72^ Although the T-cell response has not been particularly analyzed here, the local, subcutaneous administration approached in the present study has not represented any acute impact on the animals. In this regard, the histopathological observation of the spleen is fully normal (Figure 4) and the modest immune response tends to be dose-dependent, although without a robust statistical confirmation (Figure 5). On the other hand, no alterations in the blood levels of several organ-damage markers have been observed (Figure 3), in agreement with the histological analyses of the spleen, kidney and liver (Figure 4). Although deeper toxicological studies should be done during the clinically oriented adaptation of protein-only secretory granules as slow delivery systems, the study presented here allows discarding any acute organic and tissue damage or immunotoxicities associated with the aggregated status of the high-dose protein depots administered through the subcutaneous route.

## Conclusions

Self-contained, self-disintegrating protein microparticles are artificial protein depots that act as convenient platforms for the slow delivery of the building block protein in a bioactive form. Despite their interest in several clinical contexts, their potential toxicity has not been so far explored. Here, we have demonstrated that these materials, upon subcutaneous administration in mice, do not show any sign of toxicity measured through body weight evolution, histological analyses of main organs, and levels of biochemical markers of hepatic and renal damage. Since the present analysis has been extended up to ∼100 mg of protein administered/kg of body weight, much over any potential clinical use in humans, we conclude that the proposed material is intrinsically biosafe and suited for use in biological interfaces. This information definitely supports its adaptation to specific applications, and it will for sure find its regulatory route to clinics.

## Abbreviations

AST: aspartate aminotransferase
ALT: alanine aminotransferase
CRE: creatinine
DTT: dithiothreitol EDTA ethylenediaminetetraacetic acid
UA: uric acid
